# αAzithromycin has enhanced effects on lung fibroblasts from idiopathic pulmonary fibrosis (IPF) patients compared to controls

**DOI:** 10.1186/s12931-020-1275-8

**Published:** 2020-01-15

**Authors:** Kristina Krempaska, Sandra Barnowski, Jacopo Gavini, Nina Hobi, Simone Ebener, Cedric Simillion, Andrea Stokes, Ronja Schliep, Lars Knudsen, Thomas K. Geiser, Manuela Funke-Chambour

**Affiliations:** 1 Department of Pulmonary Medicine, Inselspital, Bern University Hospital, University of Bern, CH-3010 Bern, Switzerland; 2 Department for BioMedical Research, University of Bern, Bern, Switzerland; 3 Graduate School for Cellular and Biomedical Sciences, University of Bern, Bern, Switzerland; 4 Department of Visceral Surgery and Medicine, Department for BioMedical Research, Inselspital, Bern University Hospital and University of Bern, 3010 Bern, Switzerland; 5 AlveoliX AG, Murtenstrasse 50, 3008 Bern, Switzerland; 6 ARTORG Center for Biomedical Engineering Research, Organs-on-Chip Technologies, University of Bern, Bern, Switzerland; 7 Bioinformatics Unit and SIB Swiss Institute of Bioinformatics, University of Bern, Bern, Switzerland; 8 Institute of Functional and Applied Anatomy, Hannover Medical School, Hannover, Germany; 9 Biomedical Research in Endstage and Obstructive Lung Disease Hannover (BREATH), Member of the German Center for Lung Research (DZL), Hannover, Germany

**Keywords:** Idiopathic pulmonary fibrosis (IPF), Anti-fibrotic drug, Azithromycin, Lysosomes, Apoptosis

## Abstract

**Background:**

Idiopathic pulmonary fibrosis (IPF) is a chronic fatal lung disease without a cure and new drug strategies are urgently needed. Differences in behavior between diseased and healthy cells are well known and drug response can be different between cells isolated from IPF patients and controls. The macrolide Azithromycin (AZT) has anti-inflammatory and immunomodulatory properties. Recently anti-fibrotic effects have been described. However, the anti-fibrotic effects on primary IPF-fibroblasts (FB) directly compared to control-FB are unknown. We hypothesized that IPF-FB react differently to AZT in terms of anti-fibrotic effects.

**Methods:**

Primary normal human lung and IPF-FB were exposed to TGF-β (5 ng/ml), Azithromycin (50 μM) alone or in combination prior to gene expression analysis. Pro-collagen Iα1 secretion was assessed by ELISA and protein expression by western blot (αSMA, Fibronectin, ATP6V1B2, LC3 AB (II/I), p62, Bcl-xL). Microarray analysis was performed to screen involved genes and pathways after Azithromycin treatment in control-FB. Apoptosis and intraluminal lysosomal pH were analyzed by flow cytometry.

**Results:**

AZT significantly reduced collagen secretion in TGF-β treated IPF-FB compared to TGF-β treatment alone, but not in control-FB. Pro-fibrotic gene expression was similarly reduced after AZT treatment in IPF and control-FB. P62 and LC3II/I western blot revealed impaired autophagic flux after AZT in both control and IPF-FB with significant increase of LC3II/I after AZT in control and IPF-FB, indicating enhanced autophagy inhibition. Early apoptosis was significantly higher in TGF-β treated IPF-FB compared to controls after AZT. Microarray analysis of control-FB treated with AZT revealed impaired lysosomal pathways. The ATPase and lysosomal pH regulator ATP6V0D2 was significantly less increased after additional AZT in IPF-FB compared to controls. Lysosomal function was impaired in both IPF and control FB, but pH was significantly more increased in TGF-β treated IPF-FB.

**Conclusion:**

We report different treatment responses after AZT with enhanced anti-fibrotic and pro-apoptotic effects in IPF compared to control-FB. Possibly impaired lysosomal function contributes towards these effects. In summary, different baseline cell phenotype and behavior of IPF and control cells contribute to enhanced anti-fibrotic and pro-apoptotic effects in IPF-FB after AZT treatment and strengthen its role as a new potential anti-fibrotic compound, that should further be evaluated in clinical studies.

## Background

Idiopathic pulmonary fibrosis (IPF) is a devastating progressive lung disease causing dyspnea and cough, which ultimately leads to respiratory failure [1]. IPF affects mainly male patients over 60 years and genetic associations have been described [2]. The mortality is comparable to severe cancer disease with a median survival of 2–3 years [2]. New drugs slow down disease progression and may prolong survival [3, 4]. Nevertheless, today no cure is available and in advanced disease eligible patients ultimately require lung transplantation, or else they succumb to their disease [2]. New and improved drug strategies are thus urgently needed.

To develop or apply existing drugs for new use, the underlying pathomechanisms need to be fully understood. The pathogenesis of IPF remains still unclear. Presently it is believed that injury of the alveolar epithelial lining followed by aberrant wound healing is the initiating event for IPF development [5]. Although epithelial cells are thought to initiate fibrosis, differentiated fibroblasts, so-called myofibroblasts, are the main mesenchymal actor cells in fibrogenesis. They originate from local fibroblasts or other precursor cells and produce extracellular matrix leading to increased lung stiffness and loss of respiratory function [6]. The differentiation into myofibroblasts is driven by pro-fibrotic mediators, including cytokines such as transforming growth factor-β (TGF-β) [7], which is considered the main pro-fibrotic cytokine [8].

Fibroblasts isolated from IPF patients show particular properties such as insufficient autophagy [9, 10]. Impaired autophagy in IPF lungs may contribute to fibrogenesis and promote fibroblast activation and extracellular matrix production [11]. Autophagy can be modulated by drugs. Recently, a study has shown that autophagy is reduced after Azithromycin (AZT) treatment and affects fibrosis [12]. Autophagy inhibition in lung fibroblasts induced proteasomal degradation of pro-fibrotic NOX4 [13]. NOX4 reduction then decreased myofibroblast differentiation in normal human lung fibroblasts [12]. In vivo, AZT has anti-fibrotic effects in the bleomycin rodent model for lung fibrosis [14]. In clinical routine AZT is used as an antibiotic. AZT is a potent macrolide antibiotic known to inhibit bacterial protein synthesis by binding to the 50S large ribosomal unit [15]. AZT shares the same mechanism of antibacterial action as other macrolides, but it has been found to accumulate more effectively in phagocytes where it can efficiently be delivered to the site of infection [15, 16]. AZT was also described to accumulate in other cell types such as epithelial cells and fibroblasts [15]. Long-term use of AZT in COPD, asthma and cystic fibrosis has been reported with improved clinical outcomes such as reduced exacerbations and cough [17–19]. The exact mechanisms remain unknown, but anti-inflammatory effects on macrophages and neutrophils might be involved [20]. Inflammation also contributes to acute exacerbations in IPF [21]. In recent retrospective clinical observations, AZT reduced mortality in acute exacerbations and hospitalization rates of IPF patients [22, 23]. In addition to antimicrobial effects, AZT might act on inflammation and thus reduce severity of acute exacerbation. Despite recent findings of a positive role of AZT in lung fibrosis, the role and precise mechanism of its effects on exacerbation and lung fibrosis remain unclear. Also, cells might act differently when derived from patients or healthy controls [24].

In our study, we tested the anti-fibrotic properties of AZT. We hypothesized that IPF fibroblasts react differently to AZT treatment than normal fibroblasts. We thus investigated whether AZT has enhanced anti-fibrotic effects on IPF fibroblasts compared to control fibroblasts. We further investigated the underlying mechanisms of divergent responses from control and IPF fibroblasts specifically for cell death, autophagy as well as lysosomal function.

## Methods

### Primary fibroblasts from IPF patients and controls

We obtained control fibroblasts from healthy lung tissue from patients undergoing tumor resection at the University Hospital of Bern, Switzerland. All study participants provided their informed written consent, as approved by the local Ethics Committee, Bern, Switzerland (KEK-BE:042/2015). We received additional lung fibroblasts from control and IPF patients from diagnostic biopsies and/or lung explants isolated and cultured as previously described and kindly provided by Prof. Crestani, Paris, France [25].

### Material, cell culture and antibodies

We used Ham’s F-12 K (Gibco, Waltham, MA) media supplemented with 10% Fetal Bovine Serum (FBS) (Gibco, Waltham, MA) and 1% Penicillin Streptomycin (Gibco, Waltham, MA) for cell culture. We treated cells for 24 h as follows: 1. Starvation media control including the respective vehicle control (Media without FBS plus 50 μM Ethanol as the dissolvent for AZT), 2. Azithromycin (50 μM, dissolved in Ethanol) (Sigma-Aldrich, St. Louis, MO) 3. TGF-β (5 ng/ml plus 50 μM Ethanol) (R&D Systems, Minneapolis, MN) or 4. Azithromycin (50 μM, dissolved in Ethanol) with simultaneous TGF-β stimulation. Each condition was tested and repeated in three independently performed experiments. The following antibodies were used for immunofluorescent staining: primary monoclonal antibody mouse anti-αSMA (Sigma-Aldrich, St. Louis, MO), monoclonal anti-vinculin-FITC (Sigma-Aldrich, St. Louis, MO) and secondary antibody Alexa Fluor 488 goat anti-rabbit (Life Technologies, Carlsbad, CA). Lysotracker Green DND-26 (Life Technologies, Carlsbad, CA) dye for life lysosomal staining. Following western blot antibodies were used: mouse anti-αSMA (Sigma-Aldrich, St. Louis, MO), rabbit anti-Fibronectin (FN) (Abcam, Cambridge, UK), rabbit anti-ATP6V1B2, rabbit anti-LC3 AB (II/I), monoclonal Smad3 and pSmad3 rabbit antibodies (Cell Signaling Technology, Danvers, MA), rabbit anti-p62 (Sigma-Aldrich, St. Louis, MO) and rabbit anti-Bcl-xL antibodies (Cell Signaling Technology, Danvers, MA). Infrared IRDye (680 or 800 CW) secondary antibodies (LI-COR Biosciences, Lincoln, NE).

### Isolation of primary control human lung fibroblasts

We isolated fibroblasts from human lung tissue, as described previously [26]. Cell culture passages between 3 and 8 were used for experiments. We treated our samples for 1 h, 24 h or 48 h as described.

### Isolation of total RNA and RT-qPCR

We used Nucleospin RNA kit (Macherey-Nagel, Switzerland) to isolate RNA according to the manufacturer’s instructions. We used the ΔΔCt method to calculate mRNA expression levels. RNA copy numbers were normalized to β2microglobulin (β2m) expression as previously described [26]. Human forward and reverse primers are listed in Table 1. Each condition was repeated three times and in independently performed experiments.

**Table 1:** Primer sequences for RT-qPCR

Gene of interest	Forward primer	Reverse primer
β2microglobulin (β2M)	CTCCGTGGCCTTAGCTGTG	TTTGGAGTACGCTGGATAGCCT
α-smooth muscle actin (αSMA)	CAGGGCTGTTTTCCCATCCAT	GCCATGTTCTATCGGGTACTTC
Collagen 1A1 (COL)	CCAGAAGAACTGGTACATCAGCA	CGCCATACTCGAACTGGGAAT
Fibronectin (FN)	TAAAGGACTGGCATTCACTGA	GTGCAAGGCAACCACACTGAC
ATP6V1B2	TAGTTCAGGTATTTGAAGGGAC	GGTGTTCGGAGAATATCCC
ATP6V0D2	TCTCACCTATATGACGTGCAGT	GGTGGCACTTCCCCAGAATTT
RAB7B	GGCCAGCATCCTCTCCAAGATTATC	GATGCAGCCATCGGAGCCCTTGT
TMEM55b	GGTTATCTGTGGACATTGCA	ATAGATGACACTTTCCTGCAG
Cathepsin B (CTSB)	TTCTTGCGACTCTTGGGACTTC	TGACGAGGATGACAGGGAACTA
Cathepsin C (CTSC)	CCTATCTTGACCTGCTGGG	CTTGTGGTCCCATAACCGA
Cathepsin D (CTSD)	AACTGCTGGACATCGCTTGCT	CATTCTTCACGTAGGTGCTGGA

### Immunofluorescent staining and microscopy

We seeded control and IPF fibroblasts on IBIDI® chamber slides (Thermo Fischer Scientific, Waltham, MA). The cells were grown until 80% confluency was reached and kept in resting media for 24 h. Afterwards, the cells were fixated and incubated with the respective primary and secondary antibodies. To identify lysosomes, we used Lysotracker® Green dye (Life Technologies, Carlsbad, CA) life staining. Pictures were taken with Leica DMI4000 B fluorescence microscope using the 20x and 63x magnification (Plan-Apochromat 63x/0.7 Oil).

### Western blot

We treated control and IPF fibroblasts with TGF-β (5 ng/ml) and/or AZT (50 μM) for 48 h for the analysis of αSMA, Fibronectin, ATP6V1B2, LC3 AB (II/I) and p62. Incubation time for Bcl-xL analysis was 24 h and 1 h to evaluate SMAD3 phosphorylation. Cells were harvested using Pierce IP Lysis Buffer® (Thermo Fischer Scientific, Waltham, MA) according to the manufacturer’s instructions. We normalized infrared fluorescent signals to internal control monoclonal β-actin mouse antibody (LI-COR Biosciences, Lincoln, NE) and quantified their intensities as previously described [26].

### Apoptosis and cell death measurements

Fibroblasts were grown to 80% confluency on 6-well plates preceding treatment. We used Annexin V (A) and Propidium Iodide (PI) (Biolegend, San Diego, CA) dyes according to manufacturer’s instructions for flow cytometry analysis. To evaluate cytotoxicity of Azithromycin in control and IPF fibroblasts, we measured the release of lactate dehydrogenase (LDH) in cell supernatants after 24 h of treatment by a colorimetric assay according to the manufacturer’s instructions. We measured the optical densitiy at 540 nm using an Infinite M1000 PRO microplate reader (TECAN, Männerdorf, Switzerland) as previously described [27]. Bcl-xL was measured by western blot as described above.

### Lysosomal pH measurements

We determined lysosomal pH using FITC-Dextran loaded fibroblasts following a previously published protocol [28]. We seeded control and IPF fibroblasts in 6-well plates with a FITC-Dextran loaded medium solution (0.1 mg/ml – FD40S – Sigma-Aldrich) for 48 to 72 h prior to treatment. FITC-Dextran medium was then removed by aspiration and fresh cell culture medium was added with the respective treatments for a 24 h incubation time. We determined the lysosomal pH by flow cytometry using a standard curve scale (pH scale from 4 to 6) which was compared to the treated samples.

### Elisa

We used a monoclonal ELISA kit (R&D Systems, Minneapolis, MN) according to the manufacturer’s instructions to determine concentrations for pro-collagen Iα1 (pro-Col1A1).

### Transmission electron microscopy

AZT-induced vacuolar structures of control and IPF fibroblasts were analyzed in Hannover, Germany at the Institute for Anatomy by transmission electron microscopy. The preparation for electron microscopic evaluation was performed according to established methods [29]. In brief, cell pellets were fixed by immersion using a fixation mixture of 1.5% PFA, 1.5% GA in 0.15 M HEPES buffer. Afterwards, fixed cells were embedded in epoxy resin (Epon®) and ultrathin sections of a thickness of 60 nm were cut. Sections were investigated using a transmission electron microscope (Morgagni, Fei, Eindhoven, The Netherlands).

### GeneChip microarray assay and data analysis

We performed microarray analysis to screen involved pathways after AZT treatment in control lung fibroblasts. Microarray analysis was performed on isolated total RNA samples. RNA isolation is described above and concentration was determined spectrophotometrically using Nanodrop (NanoDrop 2000). Sample preparation for microarray hybridization was carried out as described in the Affymetrix GeneChip WT PLUS Reagent Kit User Manual (Affymetrix, Inc., Santa Clara, CA, USA). In brief, 100 ng (50 ng of sample no. 09) of total RNA was used to generate double-stranded cDNA. 15 μg of subsequently synthesized cRNA was purified and reverse transcribed into sense-strand (ss) cDNA, whereat unnatural dUTP residues were incorporated. Purified ss cDNA was fragmented using a combination of uracil DNA glycosylase (UDG) and apurinic/apyrimidinic endonuclease 1 (APE 1) followed by a terminal labeling with biotin. 3,8 μg fragmented and labeled ss cDNA were hybridized to Affymetrix Clariom S human arrays for 16 h at 45 °C in a GeneChip hybridization oven 640. Hybridized arrays were washed and stained in an Affymetrix Fluidics Station FS450, and the fluorescent signals were measured with an Affymetrix GeneChip Scanner 3000 7G. Fluidics and scan functions were controlled by Affymetrix GeneChip Command Console v4.1.3 software. Sample processing was performed at an Affymetrix Service Provider and Core Facility, “KFB - Center of Excellence for Fluorescent Bioanalytics” (Regensburg, Germany; www.kfb-regensburg.de). RNA microarray data from control lung fibroblasts treated with Azithromycin versus non-treated can be downloaded from the ArrayExpress database at EMBL-EBI (www.ebi.ac.uk/arrayexpress) under accession number E-MTAB-8488. Summarized probe set signals in log2 scale were calculated by using the GCCN-SST-RMA algorithm with the Affymetrix GeneChip Expression Console v1.4 Software. After exporting into Microsoft Excel, average signal values, comparison fold changes and significance *P* values were calculated. Probe sets with a fold change above 2.0 fold and a student’s T-test *P* value < 0.05 were considered statistically significant. Differentially expressed genes were further analyzed and evaluated in the Department of Biostatistics, Bern, Switzerland (Cedric Simillion). Microarray data were analyzed using custom CDFs and data was normalized and log-transformed using the RMA method [30, 31]. Differential gene expression was calculated using the limma R package [32]. Pathway analysis was conducted with the SetRank package [33].

### Statistical analysis

Comparisons between control and IPF fibroblasts under different treatment conditions were tested by ONE-way ANOVA followed by Bonferroni’s multiple comparison post test for unequal sample sizes and Tukey’s post test for equal sample sizes using GraphPad Prism 7 (GraphPad Software Inc., La Jolla, CA). Student’s T-test was used to compare the mean (± SE) between only two treatment conditions. *P* < 0.05 was considered statistically significant.

## Results

### AZT has enhanced anti-fibrotic effects on extracellular matrix formation, cytokine production and myofibroblast differentiation in IPF fibroblasts compared to controls

**Fig. 1:**
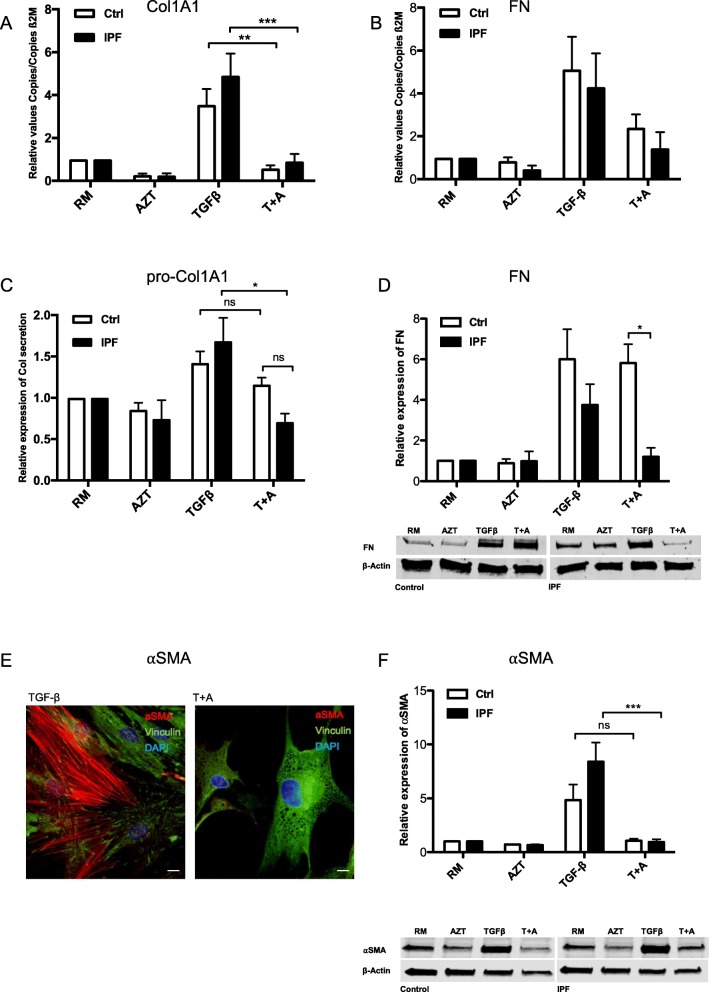
Anti-fibrotic effects of AZT in control and IPF fibroblasts. Isolated control human lung fibroblasts (*n* = 5) and IPF fibroblasts (*n* = 5) were treated with resting media (RM) for 24 h, 50 μM AZT, 5 ng/ml TGF-β or co-incubated with TGF-β + AZT (T + A). Pro-fibrotic gene expression was determined by RT-qPCR (**a**: Col1A1, **b**: FN). Data represents fold increase relative to the negative control (RM). One-way ANOVA followed by Tukey’s post test was used for statistical analysis. Protein expression was determined by ELISA (**c**: pro-Col1A1) and one-way ANOVA followed by Bonferroni’s post test was used for statistical analysis. For western blot analysis (**d**: FN; **f**: αSMA) one-way ANOVA followed by Tukey’s post was used to analyze data. **a** Col1A1 gene expression was significantly reduced in control and IPF fibroblasts after AZT and TGF-β treatment compared to TGF-β alone (**p-value ≤0.01, ***p-value ≤0.001). **b** FN gene expression was not significantly altered after AZT neither in control nor in IPF fibroblasts compared to TGF-β alone. **c** Collagen (pro-Col1A1) production was measured by ELISA in cell culture supernatant from control (*n* = 4) and IPF fibroblasts (*n* = 5). Additional AZT treatment induced a significant reduction in collagen secretion compared to TGF-β treatment alone in IPF fibroblasts, but not in control fibroblasts (*p-value ≤0.05). Combined data is presented and data was normalized to β2M, and means (+/− SEM) are presented as fold increase relative to the negative control RM. Combined data were analyzed. **d** FN protein expression was assessed by western blot analysis in whole cell lysates of control and IPF fibroblasts. FN protein expression was significantly reduced after AZT and TGF-β treatment in IPF fibroblasts when directly compared to controls (*p-value ≤0.05). Combined quantified data of control and IPF patients (*n* = 4) relative to RM are shown. **e** Immunofluorescent staining of IPF fibroblasts visually revealed that αSMA fiber expression (red) was reduced after AZT treatment compared to TGF-β alone and induced vacuolar formations. Magnification of 20x. Scale bar = 25 μM. **f** αSMA protein expression was significantly reduced only in IPF fibroblasts after AZT and TGF-β treatment compared to TGF-β alone (***p-value ≤0.001). Combined quantified data of control and IPF patients (*n* = 4) relative to RM are shown. At least three independent experiments were performed

We investigated whether Azithromycin (AZT) differently affects the fibrotic response in lung fibroblasts from normal human lung fibroblasts and IPF fibroblasts (referred to as controls and IPF-FB). We found that gene expression of the pro-fibrotic markers collagen Iα1 (Col1A1) and fibronectin (FN) were expectedly increased after TGF-β stimulation over 24 h in both cell types (Fig. 1a and b). Additional AZT treatment significantly reduced Col1A1 gene expression in control and in IPF fibroblasts (Fig. 1a). The pro-fibrotic marker FN was not significantly reduced neither in control nor in IPF fibroblasts (Fig. 1b) when combined data from cells from different individuals were analyzed. However, when the individual control and IPF fibroblasts were analyzed, FN was also significantly reduced after AZT and TGF-β treatment (Additional file.1: Figure S1A and S1B). This statistical discrepancy was due to the high inter-individual variability of the TGF-β treatment response we observed in primary lung fibroblasts. In control fibroblasts pro-Col1a1 protein expression was not reduced after addition of AZT, while IPF fibroblasts showed a significant reduction compared to controls (Fig. 1c). Similar, no reduction was seen for FN protein expression in control fibroblasts after AZT in addition to TGF-β, but FN protein expression was significantly reduced in IPF fibroblasts compared to controls (Fig. 1d). We speculate that differences between gene and protein expression might be influenced by enhanced post-transcriptional effects of AZT in IPF fibroblasts compared to controls. Last, we analyzed the myofibroblast differentiation marker αSMA by qPCR, western blot and immunofluorescent staining. AZT treatment in addition to TGF-β treatment significantly reduced αSMA in control and IPF fibroblasts on gene expression level (Additional file 1: Figure S1C). Combination treatment with AZT showed a visual reduction of actin fibers in both control (not shown) and IPF fibroblasts (Fig. 1e). Notably, we observed fortuitously vacuolar formations after AZT treatment in most of control (not shown) and IPF fibroblasts in immunofluorescence staining for αSMA (Fig. 1e). Protein expression of αSMA was only significantly decreased in IPF fibroblasts after additional AZT treatment (Fig. 1f). IPF fibroblasts are considered to be more sensitive towards TGF-β treatment [34]. With regards to αSMA protein expression, we could observe enhanced TGF-β responsiveness in IPF fibroblasts compared to controls, although not statistically significant in our experiment (Fig. 1f).

### Azithromycin interferes with autophagy in control and IPF fibroblasts

**Fig. 2:**
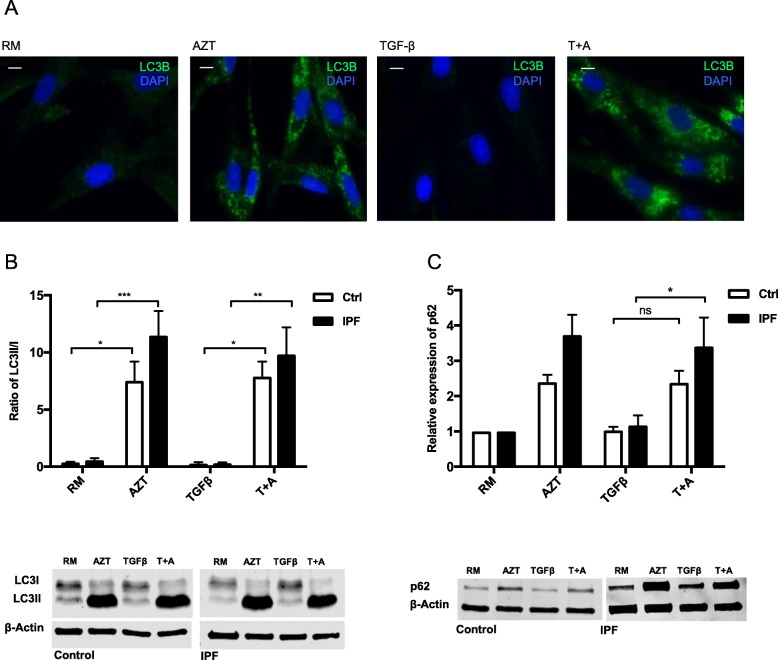
Azithromycin interferes with autophagy in control and IPF fibroblasts. **a** Immunofluorescent staining of control fibroblasts visually revealed LC3B positive cells (green) after AZT treatment compared to TGF-β or RM alone (*n* = 2). Magnification of 20x. Scale bar = 25 μM. **b** Western blot analysis of LC3I and LC3II was performed on control and IPF fibroblast lysates after 48 h treatment. One representative example is shown. Ratio of LC3II/I, normalized to β-Actin, showed a significant increase after AZT alone or in combination with TGF-β in both control (white bars, *n* = 4) and IPF fibroblasts (black bars, *n* = 4, *p-value ≤0.05, **p-value ≤0.01, ***p-value ≤0.001). **c** Quantification by densitometry of p62 relative to RM showed a significant increase after AZT and TGF-β treatment in IPF fibroblasts (black bars, *n* = 4, *p-value ≤0.05), but not control fibroblasts. One representative example of each western blot is shown and at least three independent experiments were performed. One-way ANOVA followed by Tukey’s post test was used for statistical analysis

Vacuolar formations in Fig. 1e suggest that AZT might interfere with lysosomal and/or autophagic processes. We thus performed an LC3B immunofluorescent staining as a marker for autophagosomes. As shown in Fig. 2a, AZT induced an accumulation of LC3B positive cells in control fibroblasts compared to AZT non-treated cells (RM and TGF-β). We further analyzed the protein expression of the autophagic markers LC3I, LC3II and p62. AZT treatment increased the LC3II/I ratio as a measurement of autophagic flux with and without TGF-β in both control and IPF fibroblasts (Fig. 2b). Increased ratio of LC3II/I indicates decreased autophagic flux and thus decreased autophagic activity [35]. For confirmation, we performed p62 western blot analysis which serves as a selective substrate for autophagy and is accumulated upon autophagy inhibition. While increase of p62 protein expression was not significant in control fibroblasts after combined AZT and TGF-β treatment, IPF fibroblasts had a significant increase in p62 protein expression, and thus more autophagy reduction than controls (Fig. 2c).

### Azithromycin enhanced early apoptosis in IPF fibroblasts compared to controls

**Fig. 3:**
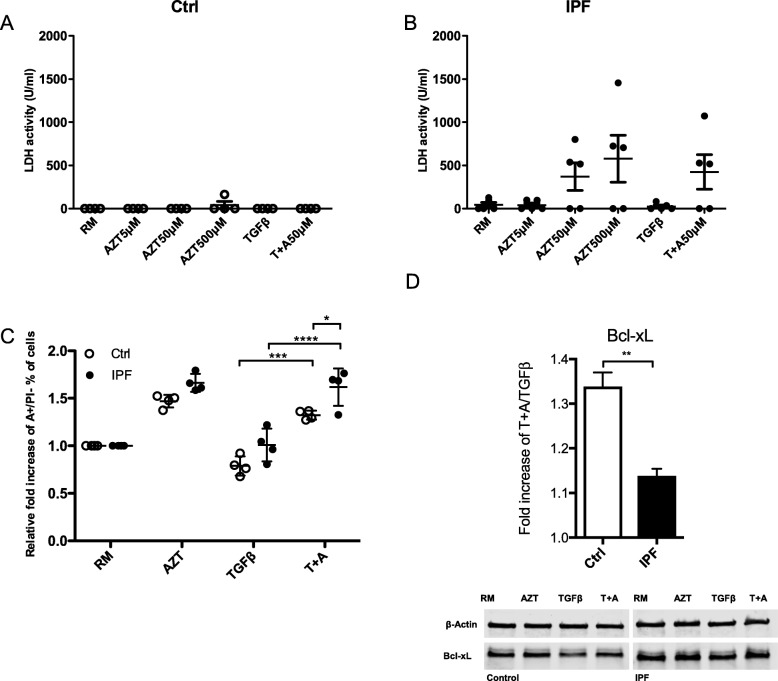
Azithromycin enhanced early apoptosis in IPF fibroblasts compared to controls. **a** No cytotoxicity was measured by LDH release in cell supernatant in control fibroblasts (*n* = 4). **b** Most IPF fibroblasts (*n* = 5) showed increased cytotoxicity after AZT (50 μM and 500 μM) treatment in the presence or absence of TGF-β. No statistical difference was reached. **c** Significant increase of early apoptosis (A+/PI-) in both control (*n* = 4, ***p-value ≤0.001) and IPF fibroblasts (*n* = 4, ****p-value ≤0.0001) is observed after co-stimulation with TGF-β and AZT. IPF fibroblasts had significantly higher early apoptosis level after costimulation compared to controls (*p-value ≤0.05). Data represent fold increases relative to the negative control (RM). One-way ANOVA followed by Tukey’s post test was used for statistical analysis. **d** Bcl-xL protein expression was assessed by western blot analysis in whole cell lysates from control or IPF fibroblasts. Bcl-xL protein expression was significantly reduced after additional AZT treatment in IPF fibroblasts when directly compared to controls (paired students t-test; **p-value ≤0.01). Normalized fold decrease of Bcl-xL expression of TGF-β and AZT relative to TGF-β is shown (*n* = 3). At least three independent experiments were performed

To determine AZT cytotoxicity, we measured LDH release from cell supernatants after treatment. No cytotoxicity was observed in control fibroblasts treated with increasing concentration of AZT (Fig. 3a). Interestingly, we observed increased LDH levels in most but not all IPF fibroblasts after AZT with or without TGF-β (Fig. 3b). No statistical difference could be detected due to inter-individual variability. Our results suggest that some IPF fibroblasts are more sensitive to AZT treatment than control fibroblasts. To further analyze AZT-induced cell death and apoptosis, we performed Annexin V/PI staining measured by flow cytometry. Both control and IPF fibroblasts showed increased early apoptosis defined as Annexin positive and PI negative signal after co-stimulation with AZT and TGF-β compared to TGF-β alone. When compared to controls, AZT and TGF-β induced significantly higher early apoptotic levels in IPF fibroblasts (Fig. 3c). In addition, we performed Bcl-xL western blot analysis and observed a decrease in IPF fibroblasts compared to controls after TGF-β and AZT (Fig. 3d), indicating reduced anti-apoptotic activity by Bcl-xL.

### Azithromycin alters several genes involved in lysosomal pathways

**Fig. 4:**
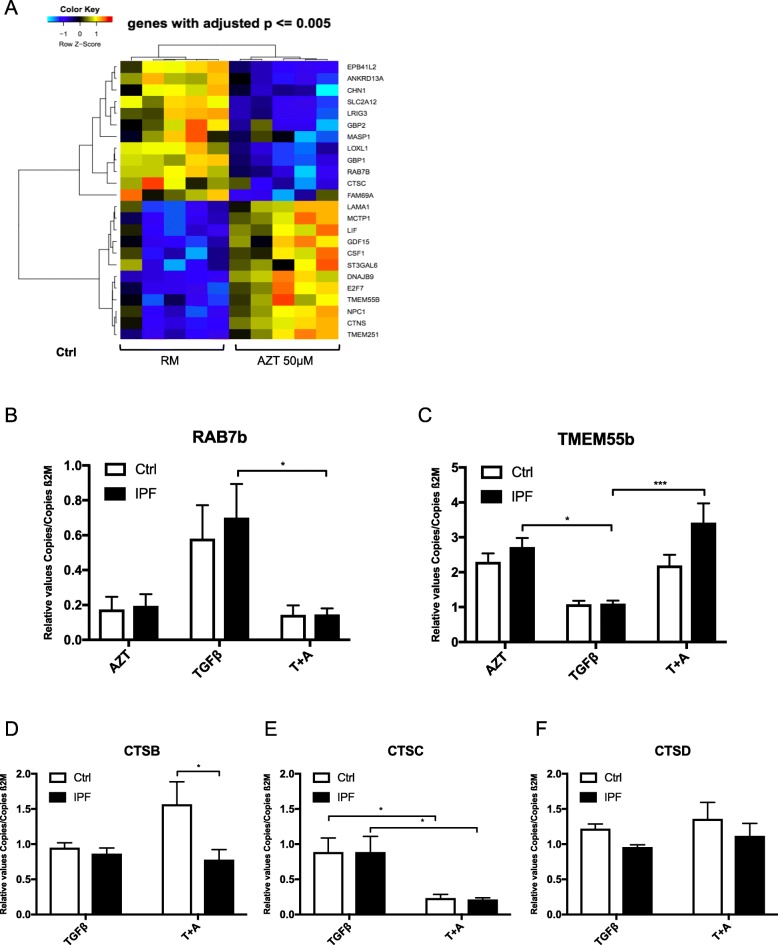
Azithromycin alters genes involved in lysosomal pathways. **a** Heat map of microarray analysis in primary control lung fibroblasts (*n* = 5) is shown. Presented are the 24 most differently regulated genes (*p* ≤0.005). Several genes are involved in lysosomal pathways. **b**, **c**: To compare control and IPF fibroblasts, RAB7b and TMEM55b were analyzed by qPCR. **b** RAB7b was significantly more downregulated after additional AZT (T + A) compared to TGF-β alone in IPF fibroblasts but not in controls (*n* = 4) (*p-value ≤0.05). **c** TMEM55b was significantly upregulated after additional AZT (T + A) compared to TGF-β alone in IPF fibroblasts but not in controls (*n* = 4) (*p-value ≤0.05, ***p-value ≤0.001). **d**-**f** Cathepsin **b**, **c** and **d** were analyzed by qPCR comparing control and IPF fibroblasts. **d** Cathepsin **b** was significantly higher upregulated after additional AZT in control fibroblasts compared to IPF (*p-value ≤0.05). **e** Cathepsin C was significantly downregulated in control and IPF fibroblasts after additional AZT (T + A) compared to TGF-β alone (*p-value ≤0.05). **f** Cathepsin D was not significantly altered after additional AZT (T + A) compared to TGF-β. All experiments were performed in at least three independent settings. One-way ANOVA followed by Tukey’s post test was used for statistical analysis

To further elucidate the involved mechanisms of AZT effects, we performed microarray screening of control lung fibroblasts with and without AZT treatment. Microarray analysis showed the engagement of several lysosomal genes that were either up- or downregulated (Fig. 4a). A heat-map from microarray analysis with the 24 most differently regulated genes (*p* < =0.005) (Fig. 4a) indicated that AZT treatment mostly impairs lysosomal genes. RAB7B, which is among others responsible for transportation of endosomes to the Golgi network [36] as well as degradation of proteins in lysosomes, [37] was downregulated after AZT treatment. Gene expression analysis revealed a greater decrease after TGF-β and AZT in IPF compared to controls of RAB7B if compared to TGF-β stimulation alone (Fig. 4b). The lysosomal membrane marker TMEM55b, which was upregulated in our microarray analysis, was more increased on gene expression level after additional AZT treatment in IPF fibroblasts compared to controls (Fig. 4c). We further analyzed Cathepsin B (CTSB) and found a significant increase of CTSB after additional AZT treatment in control compared to IPF fibroblasts (Fig. 4d). Cathepsin C (CTSC), a lysosomal cysteine proteinase, [38] was downregulated after additional AZT treatment in control and IPF fibroblasts (Fig. 4e). Last, we analyzed gene expression of Cathepsin D (CTSD) and did not see any significant changes after treatment (Fig. 4f).

### Azithromycin induces lysosomal accumulation in control and IPF fibroblasts

**Fig. 5:**
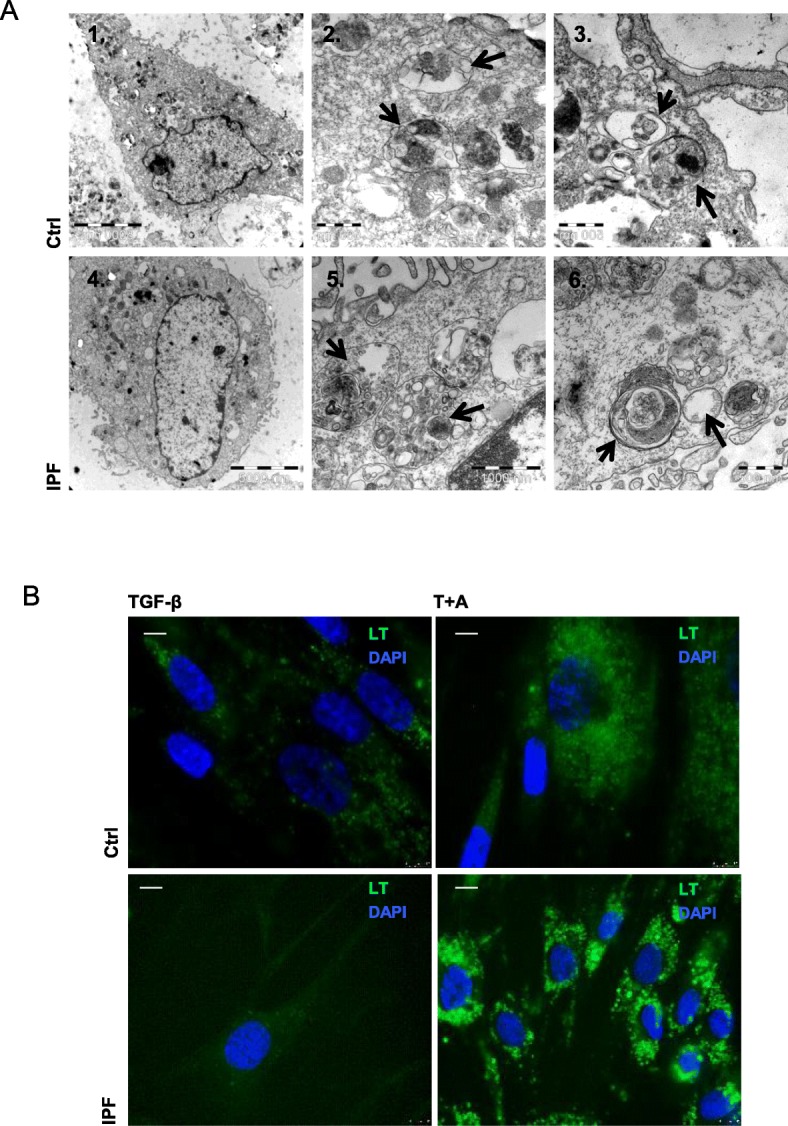
Azithromycin induces lysosomal accumulation in control and IPF fibroblasts. **a** Control (A1–3) and IPF fibroblasts (A4–6) were treated with AZT for 24 h, fixed and analyzed by electron microscopy. (A1, 4) Overview, (A2, 5) late endosomes or lysosomes (arrows), (A3, 6) autolysosomes, double membrane (arrows). **b** Lysotracker green staining in control (upper images, *n* = 3) and IPF fibroblasts (lower images, *n* = 3) after 24 h treatment with AZT in presence or absence of TGF-β confirmed the vacuolar structures as lysosomes. Magnification of 63x. Scale bar = 10 μM. All experiments were performed in three independent experiments

AZT-induced vacuolar structures of control and IPF fibroblasts were analyzed in Hannover, Germany at the Institute for Anatomy by electron microscopy (EM). The vacuolar structures were identified in control (Fig. 5a1–3) and IPF fibroblasts (Fig. 5a4–6) as late endosomes, lysosomes (Fig. 5a2 and 5) or autolysosomes (Fig.5a3 and a6). Lysosomal staining with LysoTracker (LT) green dye confirmed EM imaging finding. Lysosomes stained by LysoTracker green dye were clearly visible after AZT with or without TGF-β treatment in both control and IPF fibroblasts (Fig. 5b). Lysosomal accumulation suggests that AZT might interfere with lysosomes and lysosomal function.

### Azithromycin affects vacuolar ATPases and impairs lysosomal pH

**Fig. 6:**
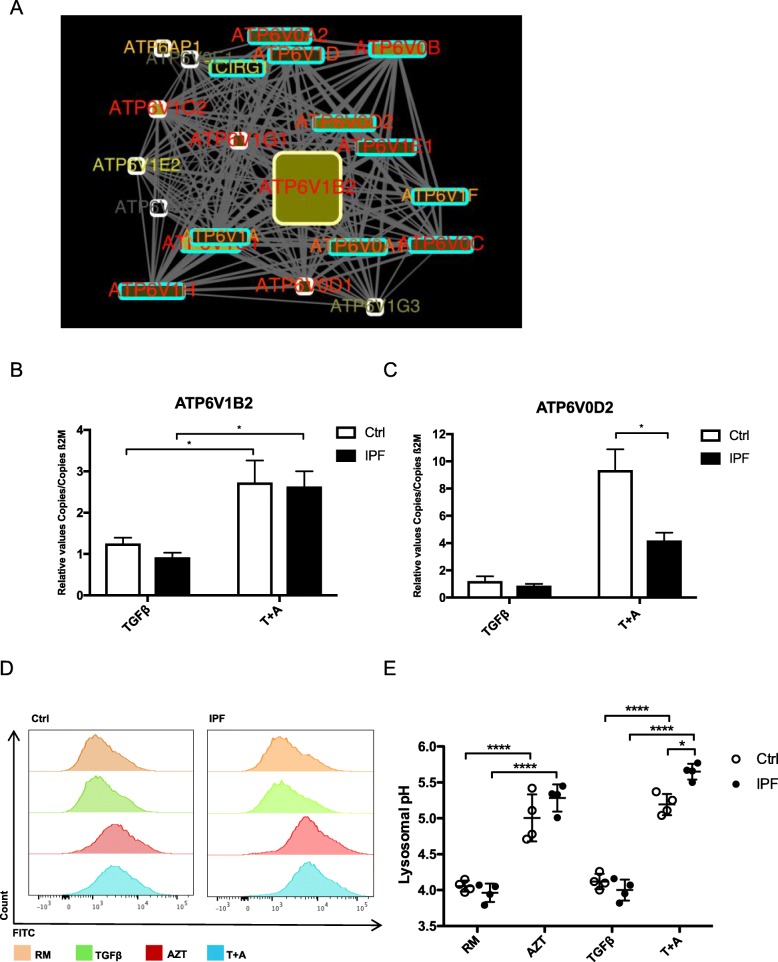
Azithromycin affects vacuolar ATPases and impairs the lysosomal pH. **a** Microarray analysis revealed the engagement of various vacuolar ATPases. Particularly ATP6V1B2 gene expression was increased, which suggests the involvement of lysosomal acidification. **b** ATP6V1B2 gene expression was significantly upregulated in control and IPF fibroblasts after additional AZT (T + A) compared to TGF-β alone (*p-value ≤0.0, *n* = 4), although no difference was observed for ATP6V1B2 between controls and IPF-FB. **c** ATP6V0D2 gene expression was significantly higher upregulated in controls after additional AZT (T + A) compared to IPF (*p-value ≤0.05, *n* = 4). **d** A representative histogram of control and IPF treated fibroblasts measured by flow cytometry is shown. Notably, a shift of the lysosomal pH represented by the FITC signal is seen after AZT treatment in both control and IPF fibroblasts and slightly stronger in the IPF fibroblasts. Combined data (*n* = 4, each) from independent experiments are shown in **e** AZT in the presence or absence of TGF-β induced a significant increase of the lysosomal pH in both control (*n* = 4, ****p-value ≤0.0001) and IPF fibroblasts (*n* = 4, ****p-value ≤0.0001). Lysosomal pH was significantly different between control and IPF fibroblasts after additional AZT (*p-value ≤0.05). All experiments were performed in three independent settings. One-way ANOVA followed by Tukey’s post test was used for statistical analysis

Microarray analysis (Fig. 4a) revealed the engagement of various vacuolar ATPases in control fibroblasts after AZT treatment as illustrated in Fig. 6a. Particularly ATP6V1B2 gene expression was increased, which is a proton pump important for lysosomal acidification and influences its function [39]. ATP6V1B2 gene expression was significantly upregulated after additional AZT compared to TGF-β alone, but no significant difference was observed between control and IPF fibroblasts (Fig. 6b), neither on protein level (Additional file 1: Figure S3). We thus analyzed additional ATPases and found that gene expression of ATP6V0D2 was significantly less upregulated after additional AZT in IPF fibroblasts compared to controls (Fig. 6c). Last, we analyzed ATPase activity and function by measuring the lysosomal pH. We observed a significant lysosomal pH increase after additional AZT treatment in control and IPF fibroblasts compared to TGF-β alone. However, IPF fibroblasts had a significantly higher increase of pH and thus more impaired function after additional AZT when directly compared to control fibroblasts (Fig. 6d and e).

## Discussion

Our study shows that AZT has anti-fibrotic and pro-apoptotic effects on primary fibroblasts and that these effects are enhanced in IPF fibroblasts compared to control fibroblasts. We confirmed that AZT reduces gene expression levels of pro-fibrotic genes after stimulation with TGF-β in vitro in control as well as in IPF fibroblasts as recently reported [12]. In our study, AZT impairs lysosomal pH and autophagy in both, IPF and control fibroblasts but effects are enhanced in cells from IPF patients. Our study shows for the first time that AZT has enhanced anti-fibrotic effects on collagen, fibronectin, αSMA and pro-apoptotic markers in IPF fibroblasts compared to controls and that impaired lysosomal function might contribute to this finding. Our results further support the role of AZT as a potential treatment for IPF.

Studies with antimicrobial therapies have been previously proposed for IPF treatment as increased bacterial burden in the BAL of IPF predicted lung functional decline [40]. Preventive antibiotic treatment with Cotrimoxazole in IPF showed decreased mortality and lower oxygen needs [41]. However, effects of antibiotics, specifically macrolides, might go beyond the scope of its antibiotic effects and additional effects might even indirectly or directly alter disease course in IPF. Next to antimicrobial properties, AZT is known to reduce gastrointestinal reflux and aspiration [42]. Reflux is thought to contribute to IPF pathogenesis [43]. The acidic material of the reflux increases epithelial permeability and as a consequence promotes fibrogenesis [43]. Moreover, direct anti-fibrotic effects of AZT have been previously described in the bleomycin mouse model of lung fibrosis. Wuyts et al. showed that collagen deposition and spindle cell proliferation were reduced after AZT treatment [14]. AZT has been described to highly accumulate in tissues and fibroblasts [44] achieving up to 100-fold higher concentrations than those in plasma [15]. This accumulation might even amplify the anti-fibrotic effects of AZT on fibroblasts in the fibrotic lung. Anti-fibrotic effects have also been found with other macrolides. A derivative of Erythromycin, EM703, was able to reduce the transcription of collagen in normal and scleroderma fibroblasts [45]. EM703 also downregulated collagen in the bleomycin mouse model by modulating TGF-β signaling in lung fibroblasts, suggesting a potential role for macrolides in fibrosis treatment [46]. While anti-fibrotic effects have been described for macrolides, the effects in diseased and healthy cells remain unexplored. Different behavior of primary diseased cells and healthy controls is commonly observed [24]. It has been found that IPF fibroblasts are more resistant to collagen matrix induced cell death compared to controls [47]. Vuga et al. found that upregulation of the WNT5A gene was responsible for apoptosis resistance in fibroblasts from fibrotic lungs compared to controls [48]. Moreover, it has been shown that IPF fibroblasts have decreased FoxO3a expression causing low autophagy levels [49]. In IPF, decreased autophagy marker levels are already observed at baseline [9]. Interestingly, autophagy induction is considered anti-fibrotic and the autophagy inducer rapamycin protects against bleomycin induced lung fibrosis [11]. Our present findings are intriguing and indicate that also inhibition of autophagy by AZT has anti-fibrotic effects [12]. TGF-β the main pro-fibrotic marker driving lung fibrosis has been shown to induce autophagy in lung fibroblasts [50]. These controversial findings raise the question of which approach would be most beneficial in IPF patients: An increase or a reduction of autophagy? Today, the role of autophagy on fibrosis remains unclear and our study is unable to fully answer this question. To maintain proper cell function a balanced autophagic flux is crucial. Autophagy is a cellular process that is induced in both physiological as well as pathophysiological conditions and it is essential for cell survival to maintain a fine balance between protein synthesis and degradation [51].

Autophagy inhibition is observed after AZT treatment and prevented myofibroblast differentiation through pro-fibrotic NOX4 suppression [12], however healthy fibroblasts have not been compared to IPF fibroblasts so far and the entire mechanism of anti-fibrotic effects of AZT is not fully understood. Our observation of intracellular vacuolar formation and impaired lysosomal function after AZT is in line with several published studies where AZT induced cytoplasmic vacuoles suggested the involvement of lysosomal function [52, 53]. Lysosomal accumulation might occur after autophagosome clearance is blocked and autophagosomal degradation is impaired.

Lysosomes are organelles with a low internal pH of 4.5–5 which is known to be regulated by vacuolar ATPases [54]. The main function is the degradation of cytoplasmic material, including damaged organelles, proteins and lipids [54]. Low lysosomal pH is crucial for degradation of cytoplasmic material by lysosomal hydrolases [55]. We show that AZT affects vacuolar ATPases and its function is impaired. Furthermore, AZT affects several lysosomal genes such as Rab7b. Interestingly, Rab7b was found to be crucial for actin filament organization and remodeling. Rab7b reduction prevented stress fiber formation, reduced cell migration and adhesion to fibronectin [56]. We speculate that downregulation of the lysosomal gene Rab7b could be one possible anti-fibrotic mechanism of AZT. In addition, enhanced collagen degradation through lysosomal recycling impairment was shown in renal fibrosis by inhibition of Cathepsin D [57]. Lysosomal impairment with chloroquine, an endo/lysosome inhibitor had similar effects on collagen degradation [57]. Bafilomycin is another lysosomal vacuolar ATPase inhibitor which inhibits the autophagic flux by preventing lysosomal acidification and/or blocking the fusion of lysosomes with autophagosomes [58, 59]. Interestingly, it has been found that Bafilomycin reduced collagen and fibronectin in human atrial myofibroblasts, but did not affect Smad2/3 phosphorylation [60]. In line with this finding, we and others also have found that AZT did not influence SMAD phosphorylation (Additional file 1: Figure S2) [12]. Although Bafilomycin is a widely used autophagy inhibitor acting on the lysosomal vacuolar ATPase in vitro, it is not approved for therapeutical use in humans due to acute cytotoxic effects in vivo [61]. AZT has been used for over 30 years as an antibiotic without significant adverse health effects [15]. Although AZT represents a promising future therapeutic option for fibrosis, antibiotic resistance should be considered as long-term use of AZT was recently shown to induce macrolide resistance in severe asthma patients [62].

In addition to enhanced anti-fibrotic effects of AZT in IPF-FB, we have observed increased apoptosis of fibroblasts after AZT treatment compared to control-FB. We showed that AZT induces a stronger increase of the lysosomal pH in IPF-FB, which has been proposed to lead to lysosomal membrane permeabilization and trigger the so-called lysosomal apoptotic pathway [63, 64]. Furthermore, it has been demonstrated that lysomotropic compounds are able to increase lysosomal pH and as a consequence decrease enzyme activity and proposed to impair lysosomal function [65]. Similar, a recent study has found that lysosomal pH increase with Verteporfin induced cell toxicity in malignant hepatocellular cells only [66].

Today, two drugs approved for IPF treatment (Nintedanib and Pirfenidone) are able to slow down disease progression, but adverse effects are common and no improvement can be achieved in most patients. New therapeutic treatments and treatment combinations are thus urgently needed to not only stop, but also to reverse fibrosis. AZT is a well tolerated macrolide antibiotic with tolerable side effects and could be a potential new additional therapeutic drug for IPF, but additional in vivo studies and prospective clinical trials are needed to confirm its anti-fibrotic effects.

## Conclusions

Our study shows that AZT has a greater anti-fibrotic effect regarding collagen and fibronectin secretion as well as myofibroblast differentiation in IPF compared to controls. Moreover, AZT has enhanced pro-apoptotic effects on IPF fibroblasts. The impaired lysosomal function in IPF fibroblasts after additional AZT treatment could explain cell damage with higher apoptosis levels in IPF fibroblasts. Our findings may stimulate new treatment strategies for IPF patients, but need to be tested in controlled clinical trials.

## Supplementary information

**Additional file 1.** **Figure S1.** Azithromycin reduces gene expression of fibronectin and αSMA in IPF and control fibroblasts. **Figure S2.** SMAD phosporylation is not influenced by Azithromycin in IPF and control fibroblasts. **Figure S3.** Azithromycin does not alter protein expression of ATP6V1B2 in IPF and control fibroblasts.

## Data Availability

All data generated or analyzed in this study is available from the corresponding author upon request. RNA microarray data from control lung fibroblasts treated with Azithromycin versus non-treated can be downloaded from the ArrayExpress database at EMBL-EBI (www.ebi.ac.uk/arrayexpress) under accession number E-MTAB-8488.
