# Correction to: Azithromycin has enhanced effects on lung fibroblasts from idiopathic pulmonary fibrosis (IPF) patients compared to controls

**DOI:** 10.1186/s12931-020-1304-7

**Published:** 2020-01-28

**Authors:** Kristina Krempaska, Sandra Barnowski, Jacopo Gavini, Nina Hobi, Simone Ebener, Cedric Simillion, Andrea Stokes, Ronja Schliep, Lars Knudsen, Thomas K. Geiser, Manuela Funke-Chambour

**Affiliations:** 1 Department of Pulmonary Medicine, Inselspital, Bern University Hospital, University of Bern, CH-3010 Bern, Switzerland; 2 Department for BioMedical Research, University of Bern, Bern, Switzerland; 3 Graduate School for Cellular and Biomedical Sciences, University of Bern, Bern, Switzerland; 4 Department of Visceral Surgery and Medicine, Department for BioMedical Research, Inselspital, Bern University Hospital and University of Bern, 3010 Bern, Switzerland; 5 AlveoliX AG, Murtenstrasse 50, 3008 Bern, Switzerland; 6 ARTORG Center for Biomedical Engineering Research, Organs-on-Chip Technologies, University of Bern, Bern, Switzerland; 7 Bioinformatics Unit and SIB Swiss Institute of Bioinformatics, University of Bern, Bern, Switzerland; 8 Institute of Functional and Applied Anatomy, Hannover Medical School, Hannover, Germany; 9 Biomedical Research in Endstage and Obstructive Lung Disease Hannover (BREATH), Member of the German Center for Lung Research (DZL), Hannover, Germany

**Correction to: Respir Res**

**https://doi.org/10.1186/s12931-020-1275-8**

After publication of our article [1], we have been notified that an extra alpha symbol (α) was mistakenly added at the beginning of the title.

The original article has been corrected.
