# Supplementary material for: αAzithromycin has enhanced effects on lung fibroblasts from idiopathic pulmonary fibrosis (IPF) patients compared to controls
Source: Respir Res. 2020 Jan 15;21:25. doi: 10.1186/s12931-020-1275-8 (PMC6964061; doi:10.1186/s12931-020-1275-8)
Supplement: Supplementary file 1 — Additional file 1. Figure S1. Azithromycin reduces gene expression of fibronectin and αSMA in IPF and control fibroblasts. Figure S2. SMAD phosporylation is not influenced by Azithromycin in IPF and control fibroblasts. Figure S3. Azithromycin does not alter protein expression of ATP6V1B2 in IPF and control fibroblasts. [file 12931_2020_1275_MOESM1_ESM.pptx]

## Slide 1
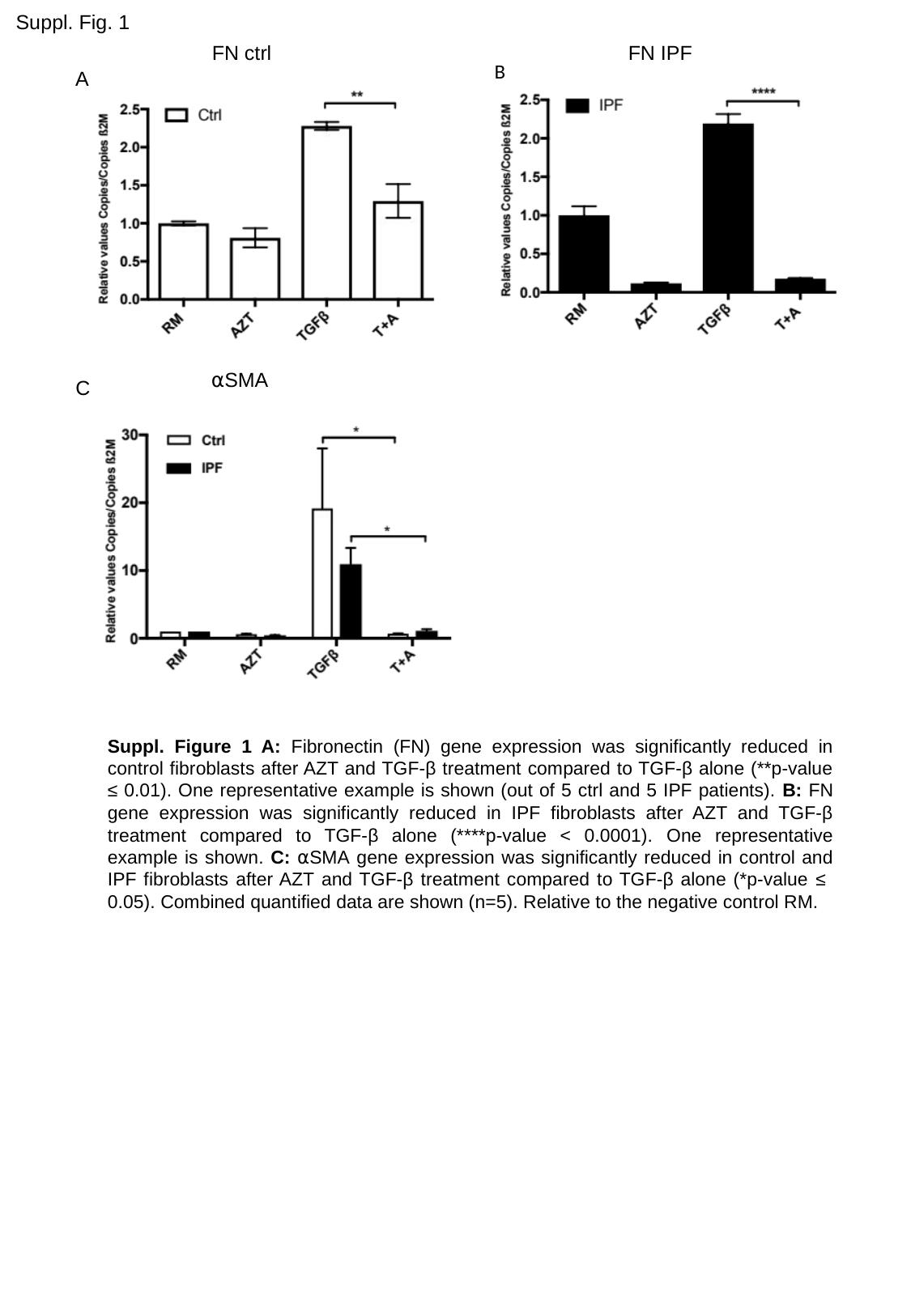

Suppl. Fig. 1
FN ctrl
FN IPF
B
A
⍺SMA
C
Suppl. Figure 1 A: Fibronectin (FN) gene expression was significantly reduced in control fibroblasts after AZT and TGF-β treatment compared to TGF-β alone (**p-value ≤ 0.01). One representative example is shown (out of 5 ctrl and 5 IPF patients). B: FN gene expression was significantly reduced in IPF fibroblasts after AZT and TGF-β treatment compared to TGF-β alone (****p-value < 0.0001). One representative example is shown. C: ⍺SMA gene expression was significantly reduced in control and IPF fibroblasts after AZT and TGF-β treatment compared to TGF-β alone (*p-value ≤ 0.05). Combined quantified data are shown (n=5). Relative to the negative control RM.

## Slide 2
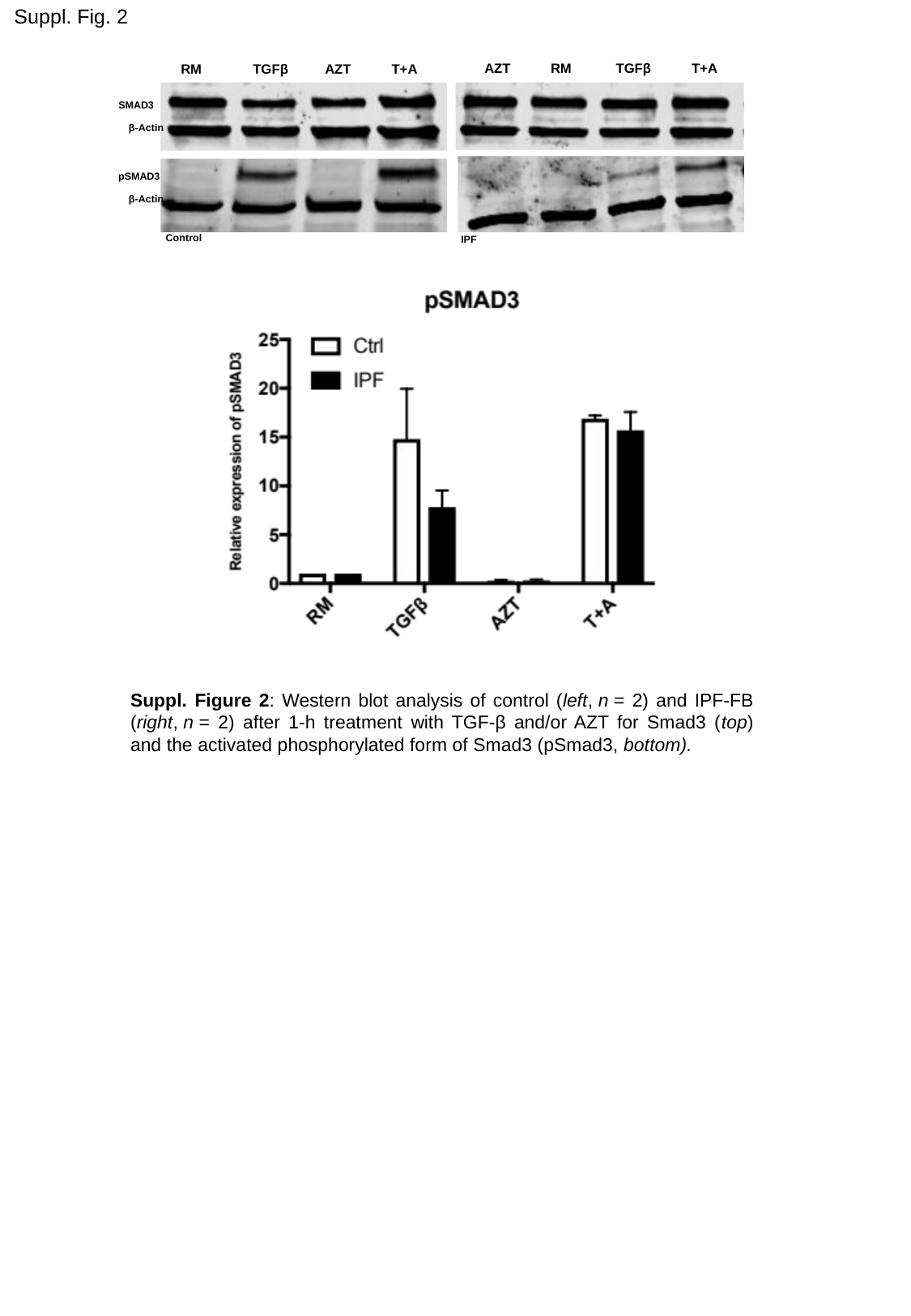

Suppl. Fig. 2
AZT
RM
TGFβ
T+A
RM
TGFβ
AZT
T+A
SMAD3
β-Actin
pSMAD3
β-Actin
Control
IPF
Suppl. Figure 2: Western blot analysis of control (left, n = 2) and IPF-FB (right, n = 2) after 1-h treatment with TGF-β and/or AZT for Smad3 (top) and the activated phosphorylated form of Smad3 (pSmad3, bottom).

## Slide 3
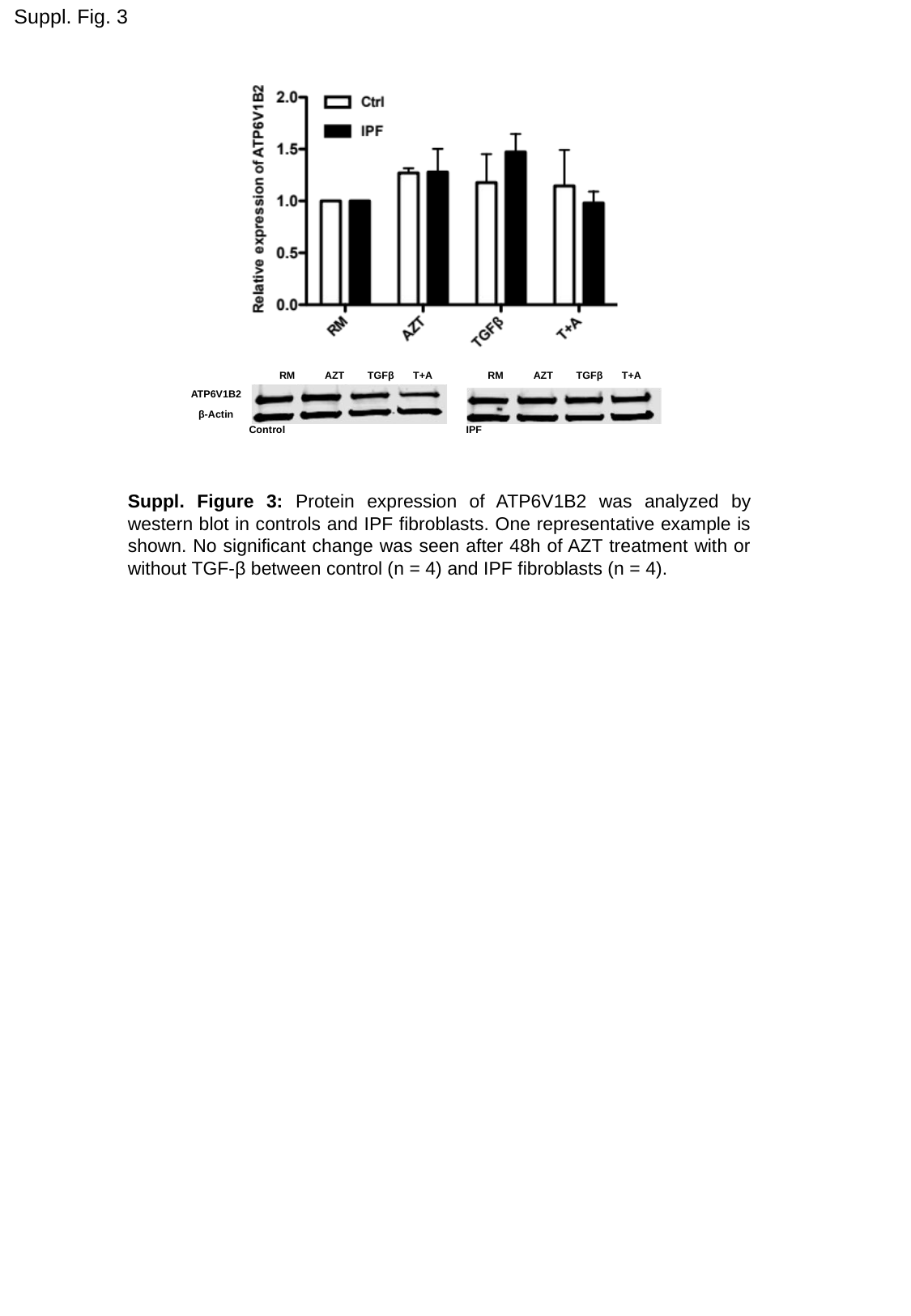

Suppl. Fig. 3
RM
AZT
TGFβ
T+A
RM
AZT
TGFβ
T+A
RM
AZT
TGFβ
TGFβ+AZT
RM
AZT
TGFβ
TGFβ+AZT
ATP6V1B2
Control
IPF
β-Actin
Suppl. Figure 3: Protein expression of ATP6V1B2 was analyzed by western blot in controls and IPF fibroblasts. One representative example is shown. No significant change was seen after 48h of AZT treatment with or without TGF‐β between control (n = 4) and IPF fibroblasts (n = 4).
